# Revolutionizing Radiological Analysis: The Future of French Language Automatic Speech Recognition in Healthcare

**DOI:** 10.3390/diagnostics14090895

**Published:** 2024-04-25

**Authors:** Mariem Jelassi, Oumaima Jemai, Jacques Demongeot

**Affiliations:** 1 RIADI Laboratory, Ecole Nationale des Sciences de l’Informatique (ENSI), Manouba University, La Manouba 2010, Tunisia; jlassi.mariem.esti@gmail.com; 2 Health Tech Innovation Systems Inc., ENSI Innovation Hub, La Manouba 2010, Tunisia; jemaioumayma98@gmail.com; 3 Ecole Supérieure des Communications de Tunis (SUP’COM), Carthage University, Ariana 2083, Tunisia; 4 AGEIS Laboratory, Faculté de Médecine, Université Grenoble Alpes (UGA), 38700 La Tronche, France

**Keywords:** automatic speech recognition (ASR), medical transcription, radiology, whisper large-v2 model, language-specific ASR systems, French language processing, AI in healthcare

## Abstract

This study introduces a specialized Automatic Speech Recognition (ASR) system, leveraging the Whisper Large-v2 model, specifically adapted for radiological applications in the French language. The methodology focused on adapting the model to accurately transcribe medical terminology and diverse accents within the French language context, achieving a notable Word Error Rate (WER) of 17.121%. This research involved extensive data collection and preprocessing, utilizing a wide range of French medical audio content. The results demonstrate the system’s effectiveness in transcribing complex radiological data, underscoring its potential to enhance medical documentation efficiency in French-speaking clinical settings. The discussion extends to the broader implications of this technology in healthcare, including its potential integration with electronic health records (EHRs) and its utility in medical education. This study also explores future research directions, such as tailoring ASR systems to specific medical specialties and languages. Overall, this research contributes significantly to the field of medical ASR systems, presenting a robust tool for radiological transcription in the French language and paving the way for advanced technology-enhanced healthcare solutions.

## 1. Introduction

The integration of Artificial Intelligence (AI) in healthcare, particularly through Automatic Speech Recognition (ASR) systems, has been a subject of increasing interest in recent years. These systems, demonstrating significant potential in various medical applications, have revolutionized the way patient–physician interactions are transcribed and clinical documentation is managed [1]. Recent explorations into online therapy platforms underscore ASR’s expanding role in mental health, where nuanced language processing enhances therapeutic outcomes [2]. In the field of radiology, where accuracy and efficiency in reporting are paramount, the application of ASR technology can be particularly transformative, offering a new paradigm in the way radiologists work and interact with diagnostic data [3]. Similarly, advancements in ASR for cochlear implants illustrate the technology’s potential to improve communication for individuals with hearing impairments, showcasing its adaptability to diverse healthcare needs [4].

Despite the global advancements in ASR technology, its application within the French medical context has been limited. This gap is further addressed by recent studies leveraging Large Language Models to refine the accuracy of ASR in medical transcription, promising more reliable patient records [5]. This gap is primarily due to the linguistic and terminological specificity required in medical ASR systems, which are often not met by general-purpose ASR tools [6]. The development of a French-language medical ASR system is thus a technological and linguistic challenge, requiring a deep understanding of medical terminologies and the nuances of spoken French in a clinical setting [7]. The need for extensive and specialized datasets, encompassing a wide range of medical terminologies, accents, and speech patterns specific to the medical profession, poses a significant hurdle [7]. While the broader application of ASR technology shows immense promise, the French radiological sector presents unique challenges such as the need for highly specialized terminologies and the accommodation of diverse French dialects and accents, which our research specifically aims to address. However, this also presents an opportunity to develop tailored solutions that can significantly benefit the medical community, particularly in non-English-speaking regions. Recent studies have shown promising results in cross-lingual applications of ASR, adapting systems to work with low-resource languages [8]. Moreover, the development of specialized ASR systems for healthcare chatbots in schools demonstrates this technology’s potential in pediatric care, contributing to early health assessments [9].

While ASR’s impact spans across multiple medical disciplines, the field of radiology in French healthcare settings remains largely untapped, marked by a critical need for customized ASR solutions. Our research directly responds to this gap by offering an ASR system that is not only linguistically and terminologically precise but also attuned to the operational workflows of French radiologists, thereby promising a significant leap towards streamlined radiological reporting and enhanced diagnostic efficiencies.

The use of advanced neural network models and language processing techniques has been explored to enhance the accuracy and reliability of medical ASR systems [10,11]. These advancements are not only technical but also encompass a broader understanding of the medical field, ensuring that the developed systems are finely tuned to the specific needs of healthcare professionals. Amidst these advancements, our study carves out a distinct niche by developing an ASR system that not only caters to the French-speaking medical community but is also intricately tailored to the nuanced demands of radiological terminology and practices in Francophone countries. Such nuanced applications of ASR, from enhancing mental health support to improving medical equipment interaction, underscore the technology’s evolving role in facilitating comprehensive and personalized care [12].

The primary objective of this research was to develop a specialized French-language ASR system, tailored for radiological applications. This system aims to facilitate radiologists in efficiently generating medical image reports, thereby enhancing the overall workflow in diagnostic procedures [13]. The novelty of this project lies in its focus on creating a dedicated ASR tool for radiology, addressing the scarcity of French-language audio datasets in the medical domain. By leveraging machine learning techniques, specifically tailored for medical jargon and radiological terms, this tool aims to provide accurate and efficient transcription services [14]. The potential of ASR in medicine is vast, ranging from automated transcriptions of medical reports to assisting in the drafting process, indexing medical data, and enabling voice-based queries in medical databases [15].

The implications of ASR technology extend beyond radiology to other medical fields. For instance, in emergency medical services, ASR has been assessed for its impact on stroke detection, showing potential for improving response times and diagnostic accuracy [16]. In long-term care for older adults, ASR models have been used to facilitate interview data transcription, saving time and resources [17]. Even in operating rooms, ASR techniques can be used to improve the dialogue between the surgeon and their human (e.g., surgical nurses) or digital (e.g., robotic arms) assistants [18,19]. Additionally, in pediatric care, ASR and voice interaction technologies have been explored for remote care management, demonstrating feasibility and effectiveness in tracking symptoms and health events [20].

Recent reviews in the field of healthcare have highlighted significant advancements in Automatic Speech Recognition (ASR) technology and its diverse applications. These advancements underscore the transformative potential of ASR in healthcare, paving the way for more efficient, accurate, and patient-centered medical practices.

A comprehensive review of state-of-the-art approaches in ASR, speech synthesis, and health detection using speech signals has shed light on the current capabilities and future directions of speech technology in healthcare [21]. This review emphasizes the growing importance of ASR in various healthcare settings, from clinical documentation to patient monitoring.

Another study explored the potential of real-time speech-to-text and text-to-speech converters, using Natural Language Grammar (NLG) and Abstract Meaning Representation (AMR) graphs, to enhance healthcare communication and documentation [22]. This technology could revolutionize how medical professionals interact with electronic health records, making the process more intuitive and efficient.

The robustness of ASR systems in noisy environments, a common challenge in medical settings, has also been a focus of recent research [23]. Enhancing the noise robustness of ASR systems is crucial for their effective deployment in diverse healthcare environments, from busy emergency rooms to outpatient clinics.

Furthermore, a systematic literature review on various techniques within the domain of speech recognition provides a comprehensive understanding of the advancements and challenges in this field [24]. This review highlights the rapid evolution of ASR technology and its increasing relevance in healthcare.

In addition to these technical advancements, the integration of ASR with patient-reported outcomes and value-based healthcare has been explored [25]. This integration signifies a shift towards more personalized and patient-centered healthcare models, where patient voices and experiences are directly captured and analyzed through advanced speech recognition technologies.

These reviews and studies collectively illustrate the significant strides made in ASR technology and its increasing applicability in healthcare. From enhancing clinical workflows to improving patient engagement, ASR technology is set to play a pivotal role in the future of healthcare delivery.

## 2. Methods

This study on developing an Automatic Speech Recognition (ASR) system tailored for radiological applications meticulously documents the methods and processes integral to this research. This section begins with a detailed description of the data preprocessing techniques and datasets foundational to the ASR system. It then describes the model selection criteria, training processes, and the deployment of the speech recognition application. The subsequent sections delve into the tasks and design of the system, followed by an outline of the evaluation metrics that quantify the performance of the system.

### 2.1. Data Preprocessing

#### 2.1.1. Data Source Selection and Collection Methodology

This research utilized a diverse array of audio content, with a primary focus on YouTube, which constituted approximately 90% of the sourced data. This was supplemented by audiobooks and podcasts. The selection strategy was driven by the need to cover a broad spectrum of radiological topics. YouTube, as a rich repository, provided access to a wealth of relevant material including radiology conferences, online courses, and medical descriptions. The integration of audiobooks and podcasts, forming about 10% of the dataset, enriched it with detailed presentations on radiological themes, ensuring a rich variety of accents and tonalities crucial for the development of a robust ASR system.

In this comprehensive approach to data collection, a multi-tiered methodology was employed. This involved systematic categorization based on human body systems, a targeted keyword analysis for each organ and imaging type, and the inclusion of diverse pedagogical voices. A critical component of the methodology was the technical extraction of audio from YouTube videos and podcasts, using sophisticated software tools to isolate the audio track from visual elements and to extract high-quality audio. This process created an audio-centric dataset, focusing on the auditory dimensions of medical instruction.

The compiled dataset boasts over 240 h of audio content, representing a vast educational repository.

#### 2.1.2. Data Collection Methodology

The methodology adopted for our exhaustive research and diverse data collection covering the entire human body was based on a methodical approach. This process revolved around a detailed exploration of various body systems, including the nervous system, circulatory system, digestive system, respiratory system, and skeletal system, among others. Each of these systems was meticulously investigated to identify the organs they comprise. This targeted and comprehensive approach facilitated the gathering of data specifically suited for radiology.

Simultaneously, each organ underwent an in-depth exploration aimed at classifying various pathologies and abnormalities diagnosable through radiographic images. This step was crucial to ensure precise and complete data collection.

To enrich the database, each consulted video was supplemented with relevant metadata. This includes vital information such as the type of imaging described in the video, whether MRI, X-ray, or another type. This step aimed to ensure sufficient diversity in imaging, considering that each imaging type is associated with specific terms. Furthermore, the metadata includes details such as the speaker’s accent and gender, the total video duration, the organ described in the video, and the body system to which the presented organs belong. This information was methodically organized and stored in a .csv file for efficient management and future reference. Table 1 details the distribution of this material among the various body systems, highlighting the dataset’s depth and scope:

Additionally, the number of hours of data collected for each speaker accent was calculated, as demonstrated in Table 2, to ensure a diverse representation of accents in the dataset.

#### 2.1.3. Transcription Generation

The transcription generation phase was a pivotal component of this study, entailing a rigorous evaluation of various transcription tools and models. This evaluation was meticulously orchestrated to not only gauge the performance of these tools but also to ascertain their alignment with the intricate requirements characteristic of radiological audio content. An extensive exploration of transcription tools was undertaken, encompassing leading-edge models such as Whisper, Conformer-CTC Large, and Wav2Vec 2.0. These models were deliberately selected for their avant-garde capabilities in speech recognition, alongside their potential applicability in the domain of medical imaging. This exploration was further augmented through the integration of Python’s SpeechRecognition library and specialized online transcription services like oTranscribe, thereby facilitating a holistic comparison between traditional transcription methodologies and contemporary machine learning-driven approaches.

To objectively evaluate the efficacy of each transcription tool, the Word Error Rate (WER) metric was employed as the principal evaluative criterion. A reference transcription was meticulously constructed for a specific 12 s audio sequence, serving as a benchmark for this assessment. The WER, recognized as a gold standard in speech recognition research, was computed utilizing Python’s Jiwer library, thus providing a quantitative foundation for the comparative analysis of transcription accuracy across various tools. This evaluative process was intricately designed to surmount the challenges inherent in transcribing medical imaging content, which is frequently imbued with specialized terminology and encompasses a spectrum of accents. As a result, the efficacy of each tool was appraised not solely on the grounds of its overall accuracy but also in terms of its adeptness in navigating the linguistic and acoustic idiosyncrasies intrinsic to the dataset.

The culmination of this exhaustive evaluation is encapsulated in Table 3, presenting a nuanced comparison of the WER scores attributed to each transcription tool. This table furnishes invaluable insights into the comparative strengths and limitations of each tool within the context of this research. The insights derived from this analysis were instrumental in guiding our selection of the most apt transcription tool for the dataset.

In the pursuit of selecting an apt transcription tool for the ASR system, a comparative analysis was conducted, centering on both the accuracy and practical applicability relative to the extensive dataset at hand. The Whisper Large-v2 model, developed by OpenAI, was identified as the optimal tool for this research endeavor. Despite the Assembly AI tool manifesting the lowest WER, its utility was constrained by a usage limitation of three hours per month, rendering it incompatible with the expansive scale of the dataset. In contrast, Whisper Large-v2 showcased a competitive WER of 0.142 and afforded the requisite flexibility for processing extensive audio inputs, accommodating sessions extending up to two hours.

**Figure 1:**
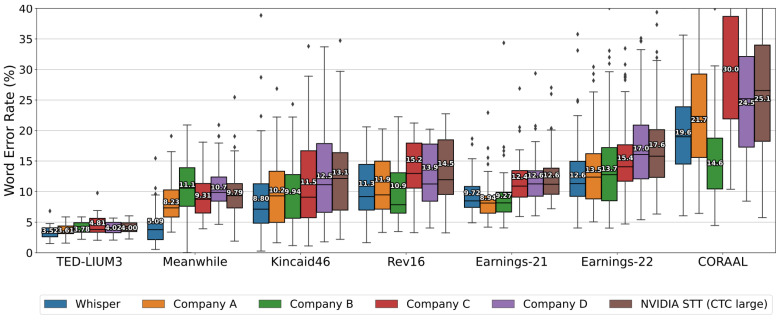
Comparative performance of the Whisper model in long-form transcription against leading ASR systems [26].

The selection of the Whisper model was underpinned by its proven adeptness in navigating diverse and challenging audio environments, a capability of paramount importance given that the dataset comprised YouTube videos and podcasts often interlaced with background noise and music. The efficacy of Whisper in such scenarios has been substantiated through rigorous comparative evaluations with leading commercial and open-source ASR systems [26]. These evaluations underscored Whisper’s superiority over the premier open-source model, NVIDIA STT, across a multitude of datasets, and its competitive stance against commercial ASR systems, thereby underscoring its adaptability and reliability across varied transcription contexts. This comprehensive performance assessment, as evidenced in Figure 1, affirmed our decision to utilize Whisper Large-v2 for the dataset’s transcription needs.

**Figure 2:**
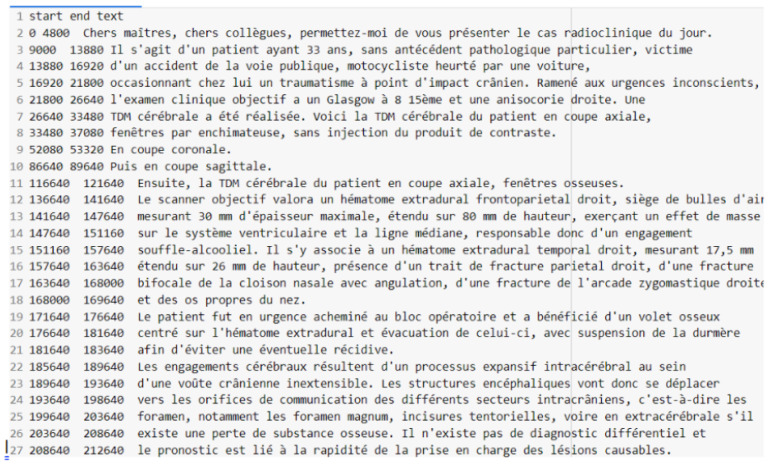
Audio file sample transcribed using Whisper Large-v2.

Figure 1 offers a visual summary of the Whisper Large-v2 model’s Word Error Rates (WERs) juxtaposed with those of other prominent ASR systems. It depicts a boxplot distribution for each system, which includes the median WER and the interquartile ranges, thereby furnishing a statistical comparison of the performance metrics. The selection of the Whisper model was substantiated by this extensive assessment, affirming its capacity to outperform the well-established NVIDIA STT model across diverse datasets and to maintain competitive accuracy alongside proprietary ASR systems. The entire dataset was transcribed using the Whisper Large-v2 model, developed by OpenAI, due to its proficiency in handling diverse audio environments and its flexibility in processing extensive inputs. This model processes audio in segments of 2 to 30 s, generating detailed transcriptions for each, which are then combined to form the transcription for the entire audio file. Upon transcription, the Whisper model generates a TSV file for each audio recording. This file details the start and end times of each sound segment and its corresponding transcription, ensuring a precise match between audio and text. The “start” and “end” columns within the TSV file demarcate the commencement and conclusion of each sequence, offering a precise temporal framework for the segmentation of both audio files and their transcriptions, as illustrated in Figure 2.

This segmentation was essential for achieving an exact match between the audio content and its textual representation. It ensured the generation of detailed and precise transcriptions for each segment, which were subsequently amalgamated to formulate a comprehensive transcription for each audio file. This methodological choice was predicated on its efficacy in managing the voluminous audio dataset and its proficiency in capturing the nuanced variations prevalent in medical dialogue.

**Figure 3:**
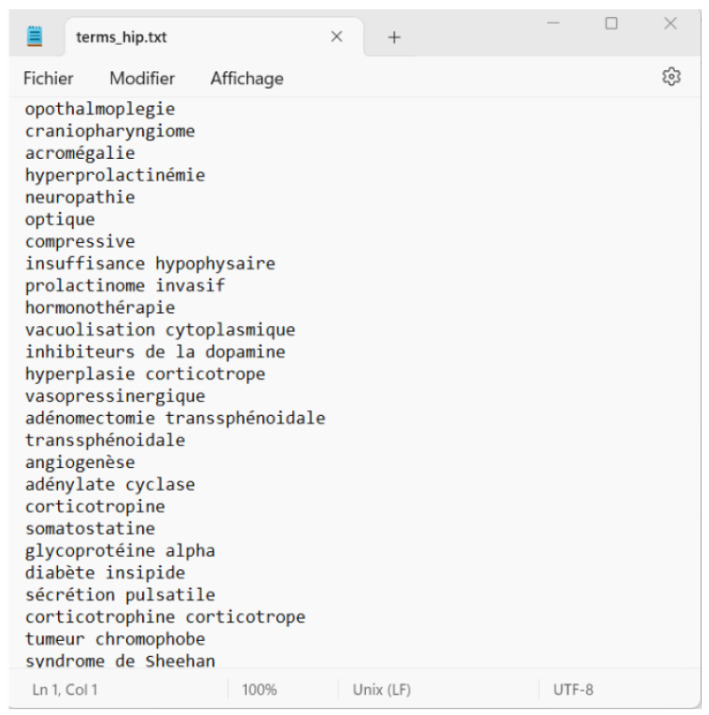
Exemplar list of technical terms.

To ensure transcription accuracy, an automated correction process was employed using the Checkspeller library, supplemented by a custom script for specialized medical terminologies. This dual approach effectively addressed both common grammatical errors and the unique challenges posed by complex medical terms. To mitigate this limitation, an integration of a specialized lexicon encompassing technical terms was undertaken, significantly enhancing the transcription accuracy, as depicted in Figure 3.

The aforementioned enhancements and rigorous verification processes underscore the meticulous approach adopted in this study to ensure the generation of high-quality accurate transcriptions. This commitment to precision laid a robust foundation for the subsequent phases of the research, particularly in the development and fine-tuning of the Automatic Speech Recognition (ASR) system tailored for radiological applications.

To demystify the transcription process and the concomitant verification mechanism with greater granularity, the subsequent Table 4 delineates the overarching methodology employed for the dataset’s transcription. This table includes a comprehensive account of the number of audio files processed, the aggregate duration of audio content, and the salient features of the transcription verification approach, thereby providing a succinct overview of the transcription methodology.

It is essential to clarify that the entire dataset underwent transcription through the selected Whisper model, which processes audio in segments from 2 to 30 s. This segmentation ensured detailed and accurate transcription for each segment, which were then cohesively assembled to form the complete transcription for each audio file. This methodology was chosen for its effectiveness in managing the extensive audio dataset and for its ability to capture the nuanced variations in medical dialogue.

Table 4 provides an overview of the transcription and verification process, detailing the total audio duration transcribed, the number of audio files processed, and the transcription verification methods employed.

#### 2.1.4. Feature Extraction

The preparation of audio data for the ASR system involved comprehensive preprocessing and feature extraction. Using the librosa library, steps such as noise reduction, silence trimming, resampling, and compression were implemented to ensure the audio quality met the transcription requirements. The process also incorporated the Whisper Feature Extractor class from the Hugging Face’s Transformers module. This stage focused on adjusting parameters like chunk length, feature size, hop length, and sampling rate, aligning them with the specific demands of the research. These modifications were essential for transforming the raw audio into a format suitable for machine learning models, laying the groundwork for model training and evaluation in this study.

### 2.2. Model Selection and Benchmarking

In developing an Automatic Speech Recognition (ASR) system tailored for radiological applications, a pivotal step involved selecting the most suitable model to ensure high transcription accuracy. Given the critical nature of radiological reports, where precision in medical terminology is paramount, the choice of the ASR model was approached with rigorous benchmarking criteria.

#### 2.2.1. Benchmarking Strategy and Datasets

The benchmarking strategy was meticulously designed to evaluate various ASR models across multiple dimensions, including the Word Error Rate (WER), computational efficiency, and adaptability to diverse speech contexts. This comprehensive approach was essential to ascertain the models’ performance in accurately transcribing radiological speech content, which is often laden with specialized terminology and presented in varied accents and background noises.

To facilitate a thorough comparison, a selection of datasets known for their relevance and complexity in speech recognition tasks was used. These included Common Voice, Multilingual LibriSpeech, VoxPopuli, African Accented French, and Fleur. Each dataset offered a unique set of challenges and speech characteristics, thereby providing a robust framework for evaluating the models’ effectiveness across a wide spectrum of real-world applications.

#### 2.2.2. Comparative Analysis and Model Selection

Our comparative analysis of ASR models, particularly focusing on the variants of the Whisper model, was grounded on a comprehensive benchmarking study utilizing datasets extracted from the Hugging Face Model Hub [27]. This approach, leveraging the platform’s extensive repository of machine learning models, provided significant insights into each model’s capabilities across a variety of speech recognition contexts. The Whisper Large-v2 model consistently outperformed its counterparts in terms of the WER across all datasets, underscoring its superior transcription accuracy. This was particularly evident in the handling of medical terminologies and in environments with diverse acoustic profiles, making it highly suitable for the project’s requirements.

For instance, in the Common Voice dataset, Whisper Large-v2 achieved a remarkable WER of 7.67%, significantly lower than the other models evaluated. Similar trends were observed across other datasets, with Whisper Large-v2 maintaining the lowest WER, affirming its exceptional performance in diverse speech recognition contexts.

These findings are encapsulated in the comprehensive benchmarking shown in Table 5, which presents a side-by-side comparison of the Whisper Large-v2 model against other leading ASR models across various datasets used in our analysis. This consolidated table underscores the consistent superiority of the Whisper Large-v2 model in terms of the Word Error Rate (WER) across all datasets, affirming its exceptional capability to meet the intricate requirements of radiological speech transcription.

This rigorous selection and benchmarking process guaranteed that the foundation of the ASR system proposed in this study rests on a model surpassing the stringent requirements of medical speech transcription, setting the stage for notable progress in radiological diagnostics.

### 2.3. Implementation

#### 2.3.1. Hardware Assessment and Initial Setup

Before embarking on the fine-tuning process, a thorough assessment of the computational resources was essential. This step is critical when dealing with advanced machine learning models like the Whisper Large-V2, which boasts 1.55 billion parameters, a testament to its complexity and capability. The hardware assessment centered on the GPU’s specifications, which play a pivotal role in a model’s training efficiency and speed.

The GPU, or Graphics Processing Unit, is a specialized electronic circuit designed to accelerate the creation and rendering of images, videos, and animations. It is also highly effective for the complex mathematical calculations often found in machine learning tasks, making it an indispensable resource in the setup.

For this study, a Tesla T4 GPU was used, known for its high performance in deep learning and AI applications. Below, Table 6 outlines the key specifications of the hardware setup.

- Tesla T4: This GPU is part of NVIDIA’s Turing family, optimized for deep learning inference. The T4 is specifically designed to accelerate diverse cloud workloads, including high-performance computing, deep learning training and inference, machine learning, data analytics, and graphics.
- GPU Memory: The 14.61 GB of memory available on the Tesla T4 GPU is crucial for handling large datasets and complex neural networks. In deep learning tasks, the GPU memory needs to accommodate the model’s parameters, gradients, and intermediate data generated during training. The Whisper model’s vast number of parameters necessitates a GPU with substantial memory to ensure smooth and efficient training.
- GPU Computing Power: Measured in TFLOPs (Tera Floating-Point Operations per Second), its computing power of 7.5 TFLOPs indicates this GPU’s capability to perform 7.5 trillion floating-point calculations per second. This high level of computational power is necessary to process the numerous operations involved in training the Whisper Large-V2 model, including matrix multiplications and other tensor operations fundamental to deep learning.

These specifications underpin the model’s training process, providing the necessary computational resources to handle the intricate tasks associated with fine-tuning a state-of-the-art ASR system like Whisper Large-V2. The robustness of the Tesla T4, combined with its ample memory and computing power, ensures that the model can be trained efficiently, paving the way for the successful implementation of the speech recognition application.

#### 2.3.2. Model Loading and Memory Optimization

To mitigate potential memory constraints, the Whisper Large-V2 model was loaded using an 8-bit precision for weights through quantization instead of 32-bit, a strategy that significantly reduced the memory footprint while preserving the accuracy of computations. This approach allowed for efficient use of the GPU memory, facilitating smoother model training sessions.

#### 2.3.3. Optimization and Fine-Tuning Strategy

In optimizing the model architecture for computational constraints, the technique of Low-Rank Adaptation (LORA) was employed. Initially, the Whisper Large-V2 model, with its 1.55 billion parameters, posed a substantial demand on computational resources. To reconcile the model’s complexity with the available hardware capabilities, a strategic reduction in trainable parameters was imperative.

Through the application of LORA, the parameter count was efficiently reduced from approximately 74.5 million to 15.7 million. This adjustment not only rendered the model more compatible with the hardware limitations but also streamlined the fine-tuning process by ensuring resource efficiency.

Text normalization was applied using the BasicTextNormalizer module, standardizing the inputs to improve the Word Error Rate (WER) evaluation consistency. This process included lowercasing, removing punctuation, and normalizing spaces.

The fine-tuning process was characterized by the strategic adjustment of training parameters, such as the batch size, learning rate, and warmup steps, optimizing the model’s performance. Table 7 presents the fine-tuning parameters and LORA configuration, for which each parameter was meticulously chosen to guide the fine-tuning process towards achieving the best possible performance from the model under the constraints of the computational resources. This careful configuration ensured that the model’s training was both effective and efficient, paving the way for its application in speech recognition tasks.

#### 2.3.4. System Pipeline and Deployment

**Figure 4:**
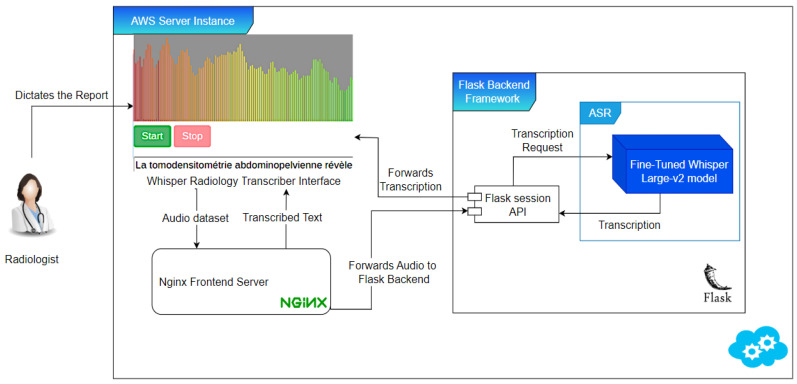
System deployment and operational flowchart.

The deployment of the “WhisperMed Radiology Transcriber”, a specialized speech recognition application tailored for radiologists, involved configuring a server environment on Amazon Web Services (AWS). Flask was utilized for back-end management, while Nginx facilitated front-end integration, as depicted in Figure 4. The architecture of this application supports audio recording through a web interface, processes the recordings on a remote server, and displays transcriptions in real-time. Designed with an emphasis on real-time transcription and an intuitive user interface, the “WhisperMed Radiology Transcriber” aims to meet the precise needs of medical professionals within French-speaking clinical settings.

## 3. Results

The comprehensive fine-tuning process of the Whisper Large-v2 model revealed significant insights into the model’s performance under various training configurations. Initially, the training parameters were meticulously configured using the Seq2SeqTrainingArguments object from the Transformers library. Key parameters such as the batch size, learning rate, and warmup steps were strategically selected to optimize the model’s performance. Notably, a learning rate of $10^{-3}$ was employed, which emerged as the most effective in enhancing the model’s accuracy, as evidenced by a notable decrease in both the Word Error Rate (WER) and Normalized Word Error Rate (Normalized WER).

In the evaluation metrics, both the Word Error Rate (WER) and Normalized Word Error Rate (Normalized WER) are reported. The inclusion of the Normalized WER offers insights into the transcription accuracy post the application of text normalization techniques. Such a metric serves as an adjusted measure, more reflective of the model’s ability to accurately decipher and transcribe medical radiology speech content. The Normalized WER evaluates the transcription’s core content fidelity, discounting variations due to punctuation, capitalization, and whitespace. This adjusted metric is crucial in a medical context where precise terminology is paramount. The observed consistent improvement in the Normalized WER over the standard WER underscores the model’s enhanced performance in transcribing detailed medical terminologies accurately.

The impact of different learning rates on the model’s performance was systematically evaluated (Table 8). Our findings indicated that a learning rate of $10^{-3}$ led to promising results, particularly with a Normalized WER of 18.774% and a Normalized Character Error Rate (CER) of 12.103%. Additionally, the application of text normalization techniques significantly reduced the WER, underscoring the importance of this preprocessing step.

Further experiments were conducted to assess the influence of warmup steps on the model’s performance. The results showed that a warmup step of 1000 yielded the best performance, achieving a Normalized WER of 18.504% (Table 9). This optimal configuration was thus subsequently adopted for further experimentation.

In addition to these parameters, this study delved into the optimization settings, particularly focusing on the Adam epsilon parameter. The optimizer’s configuration plays a crucial role in a model’s ability to converge to an optimal solution. Our experiments with different Adam epsilon values revealed significant variations in performance, as summarized in Table 10. This table illustrates how subtle changes in the optimizer settings markedly influenced the model’s effectiveness, guiding us to select the most suitable configuration for our specific needs.

The exploration of the LORA model’s “R” parameter further demonstrated the impact of model configuration on performance. The configuration with R = 42 improved the model’s performance, indicating its effectiveness in enhancing the model’s transcription accuracy (Table 11).

An additional layer of optimization focused on the Low-Rank Adaptation (LORA) technique’s dropout parameter, Lora_dropout. A systematic comparison was undertaken to determine the most effective Lora_dropout value for the model, with the objective being to optimize the balance between reducing overfitting and preserving model performance. The comprehensive results of this comparison are presented in Table 12, illustrating that a Lora_dropout value of 0.04 resulted in the most favorable performance, evidenced by a Normalized Word Error Rate (WER) of 17.121%. This optimal Lora_dropout setting contributed significantly to the model’s enhanced transcription accuracy.

The final chosen configuration, achieving a Normalized WER of 17.121%, represents the culmination of the fine-tuning efforts, incorporating the ideal Lora_dropout value. This refined configuration underscores the effectiveness of the fine-tuning strategy in enhancing the transcription accuracy for medical radiology terms.

The fine-tuning of the Whisper Large-v2 model yielded significant improvements in the transcription accuracy. This process culminated in a final Word Error Rate (WER) of 17.121%, accompanied by a training loss of 0.210 and a validation loss of 0.448.

**Figure 5:**
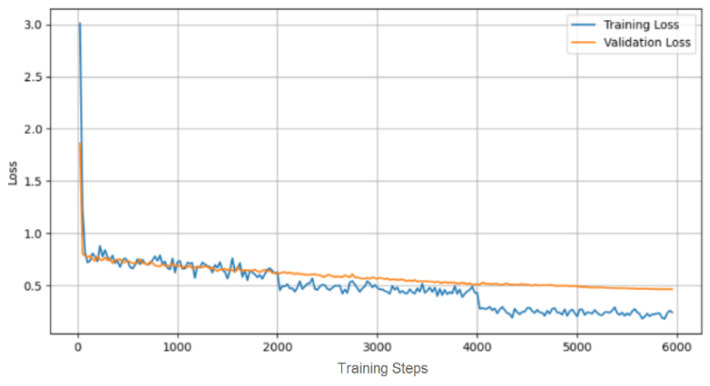
Loss curves.

These metrics demonstrate the effectiveness of the fine-tuning strategy in enhancing the model’s performance for medical radiology term transcription. The progression of the training and validation loss over the fine-tuning period is illustrated in the loss curves shown in Figure 5, providing a visual representation of the model’s learning trajectory.

Concurrently, we deployed the “WhisperMed Radiology Transcriber”, a speech recognition application, on an Amazon Web Services (AWS) server. This application utilizes the fine-tuned Whisper Large-v2 model to provide high-accuracy transcriptions of medical radiology terms. Key features of this application include real-time transcription capabilities and an intuitive user interface, designed to meet the specific needs of medical professionals.

## 4. Discussion

This study’s integration of the Whisper Large-v2 model into radiological applications marks a significant advancement in medical Automatic Speech Recognition (ASR) systems. Demonstrating high accuracy in transcribing complex medical terminology, the model’s effectiveness across diverse audio environments is a testament to its adaptability in various medical settings. This adaptability is crucial considering the acoustic complexities inherent in different medical fields. The success of AI-driven speech recognition systems in both general healthcare communication and specialized areas like radiation oncology ([28,29]) underscores their potential to revolutionize medical data processing across a spectrum of clinical contexts [30].

In clinical practices, the application of the proposed ASR system holds immense promise. The traditional process of transcribing diagnostic reports is often fraught with human error and inefficiency. By enhancing the accuracy and efficiency of medical documentation, this system stands to significantly improve the quality of patient care, as accurate records are vital for effective treatment planning [31]. Within the context of real-world application, the “WhisperMed Radiology Transcriber” stands as a Minimum Viable Product (MVP) designed specifically for radiologists. The development of this application is a direct response to the need for efficient, accurate medical transcription in radiology, aiming to minimize the time spent on generating reports while maximizing the accuracy of reports. Although the application is in its early stages, preliminary feedback from a selected group of radiologists has been promising, indicating a strong potential for integration into daily clinical practices. Future iterations of this application will focus on extensive testing with a larger cohort of medical professionals to fine-tune its functionalities and ensure seamless integration with existing hospital information systems.

Recognizing the need for a deeper analysis of our model’s performance, we acknowledge that a comprehensive error analysis, particularly focusing on different types of data within the radiological domain, would provide valuable insights into the model’s specific strengths and areas in need of improvement. Additionally, while our study highlights the model’s effectiveness in transcribing complex medical terminology, a direct comparison with established French ASR baseline models, especially those previously applied in medical contexts, remains an area for future exploration. These comparisons would not only benchmark the Whisper Large-v2 model’s performance but also pave the way for more targeted improvements, especially in handling the unique challenges presented by French medical terminology and diverse accents. Future research will aim to fill these gaps, offering a more detailed understanding of the model’s performance nuances and its standing relative to existing French ASR solutions in healthcare. Additionally, integrating ASR systems with electronic health records (EHRs) could transform healthcare data management, reducing the administrative load on medical professionals and enabling a greater focus on patient care [32].

However, the implementation of ASR in healthcare is challenging. The system must navigate a vast array of medical terminologies, accents, and speech nuances. This research represents progress in this area, but ongoing refinement is essential to meet the stringent accuracy requirements of medical data transcription [33]. Addressing these challenges, particularly in non-English languages, remains a key area for future development. Studies on language-specific medical ASR solutions, such as those in Korean and French, highlight both the challenges and opportunities in creating effective multilingual medical ASR system [28,29,30].

The “WhisperMed Radiology Transcriber” serves as the tangible outcome of this research, specifically addressing the requirements of the radiological sector. As a Minimum Viable Product (MVP), this tool seeks to enhance report generation efficiency by providing accurate medical transcriptions tailored for radiology. Initial evaluations by a select cohort of radiologists have indicated positive reception, suggesting its potential for broader applications in clinical routines. Future developments will concentrate on comprehensive real-world evaluations to refine the application, ensuring its seamless integration with existing hospital information infrastructures and compliance with stringent medical documentation standards.

Beyond its clinical applications, this ASR system offers significant benefits in medical education. By facilitating the transcription of educational materials, it enhances accessibility and inclusivity, particularly for non-native speakers. This aligns with the digitalization trend in medical education, where technology is becoming increasingly pivotal in enriching learning experiences [33].

Future research avenues are abundant. Tailoring ASR systems to specific medical specialties or languages could greatly expand their utility. Exploring their integration with voice-activated medical devices and telemedicine platforms presents opportunities to further leverage ASR technology in healthcare [34].

The current study, despite its successes, encountered limitations due to resource constraints, which restricted the dataset size and prolonged the training period. Future studies should aim to utilize larger datasets and more robust computational resources to improve accuracy and efficiency. Real-world testing in clinical settings is also crucial to assess the system’s practicality and identify areas for improvement.

The findings of this research contribute significantly to the medical ASR field, particularly in radiology transcription. The potential impact of this work on clinical practices, healthcare efficiency, and medical education underscores the vital role of technology in advancing healthcare solutions. Addressing the identified limitations, such as dataset diversity and practical application, will be essential in future research to fully realize the potential of ASR systems in healthcare.

## 5. Conclusions

This study’s development of an Automatic Speech Recognition (ASR) system specifically designed for radiological applications represents a significant advancement in the application of technology within the healthcare sector. Our successful integration of the Whisper Large-v2 model into the ASR system has led to a notable achievement: a Word Error Rate (WER) of 17.121%. This achievement underscores the system’s proficiency in accurately transcribing complex medical terminology and adapting to diverse accents, which are critical in radiological contexts.

The practical implications of this research are particularly significant in clinical settings. By automating the transcription of diagnostic reports, this ASR system addresses a key challenge in radiology—the need for accurate and efficient documentation. In light of our findings and the development of the “WhisperMed Radiology Transcriber”, this research contributes significantly to the field of medical Automatic Speech Recognition (ASR) systems. The proposed application, although currently a prototype, embodies the practical application of our research findings. It is designed to be a foundational step towards creating a more robust and comprehensive tool that can be integrated into radiology departments worldwide. Moving forward, the focus will be on expanding the application’s testing in real-world clinical environments. This will involve a series of pilot studies aimed at evaluating the application’s effectiveness in live radiological settings, thereby ensuring that the final product is both user-friendly and highly accurate, meeting the exacting standards of medical documentation.

This improvement is not just a matter of convenience; it plays a vital role in enhancing patient care by supporting informed decision making based on precise and reliable medical records.

Moreover, the potential integration of the ASR system with electronic health records (EHRs) could be a game changer in healthcare administration. Such integration promises to streamline data entry processes, reduce the administrative burden on healthcare professionals, and improve the accuracy of patient records. This aligns with the broader goal of effective healthcare delivery, where accuracy and efficiency are paramount.

While this study has achieved its primary objectives, it also highlights areas for future exploration. The potential of tailoring ASR systems to specific medical specialties or languages, and integrating them with voice-activated medical devices and telemedicine platforms [35], presents exciting avenues for expanding the utility and impact of ASR in healthcare.

Despite its successes, this study faced limitations, primarily due to resource constraints. These limitations necessitated a training dataset of 20,000 examples and extended the training period to 14 days. Future research could benefit from larger datasets and more advanced computational resources to further enhance the accuracy and efficiency of ASR systems. Real-world testing in clinical environments is also crucial to validate the practical applicability of the system and to identify areas for improvement.

In summary, this research makes a significant contribution to the field of medical ASR systems, particularly in radiology. It offers a robust and efficient tool for medical transcription, with the potential to significantly impact clinical practices and the efficiency of healthcare services. Our findings pave the way for future innovations in technology-enhanced healthcare solutions.

## Figures and Tables

**Table 1:** Distribution of audio content duration by body system.

Body System	Duration (Hours)
Nervous System	53 h 32 min
Musculoskeletal System	61 h 27 min
Endocrine System	23 h 42 min
Respiratory System	22 h 54 min
Cardiovascular System	26 h 11 min
Digestive System	19 h 43 min
Reproductive System	17 h
Urinary System	6 h 17 min
Auditory System	5 h 10 min
Lymphatic and Blood System	4 h 21 min

**Table 2:** Distribution of audio content duration by accent.

Accent	Duration (Hours)
African	16 h 33 min
Algerian	25 h 19 min
Canadian	2 h 38 min
Native French	155 h 22 min
Moroccan	29 h 17 min
Tunisian	11 h 13 min

**Table 3:** Comparative analysis of transcription tool performance based on word error rate.

Transcription Tools	WER
SpeechRecognition (Python library supporting various engines including Google Web Speech API, Sphinx, etc.)	0.228
oTranscribe (free online tool designed for audio transcription with features like playback speed control, bookmarking, etc.)	0.628
Conformer-CTC Large (voice recognition model based on the Conformer-CTC architecture, optimized for automatic speech transcription)	0.2
AssemblyAI (automatic speech transcription service using machine learning models)	0.100
Whisper Large-v2 (voice recognition model developed by OpenAI, designed for automatic speech transcription)	0.142
Wav2Vec 2.0 (model developed by Facebook AI that excels in Automatic Speech Recognition using a self-supervised learning approach)	0.257

**Table 4:** Dataset transcription and verification overview.

Attribute	Detail
Total Audio Duration	240 h and 21 min
Number of Audio Files	69,643
Transcription Verification	Automated correction with Checkspeller library and custom script for medical terms

**Table 5:** Benchmarking using various datasets.

Fine-Tuned Model	Common Voice WER (%)	LibriSpeech WER (%)	VoxPopuli WER (%)	African Accented French WER (%)	Fleur WER(%)
bofenghuang/asr-wav2vec2-ctc-french	11.440	5.130	9.330	16.220	10.100
bofenghuang/whisper-large-v2-french	7.670	4.030	8.660	4.310	4.980
jonatasgrosman/whisper-large-fr-cv11	9.087	_	_	_	8.686
bofenghuang/asr-wav2vec2-xls-r-1b-ctc-french	14.800	9.390	11.800	22.980	_
bofenghuang/whisper-medium-cv11-french	8.540	5.860	11.350	7.020	6.850
bofenghuang/deprecated-whisper-large-v2-cv11- french-punct-plus	8.030	_	_	_	5.260
bofenghuang/whisper-medium-french	8.730	4.440	9.460	4.560	5.940
bofenghuang/whisper-small-cv11-french	10.990	8.910	_	9.260	9.830
pierreguillou/whisper-medium-french	11.141	_	_	_	_
sanchit-gandhi/whisper-small-fr-1k-steps	16.998	_	_	_	_

**Table 6:** Hardware specifications.

Attribute	Detail
GPU	Tesla T4
GPU Memory	14.61 GB
GPU Computing Power	7.5 TFLOPs

**Table 7:** Fine-tuning parameters and LORA configuration for model optimization.

Parameter	Description
Num_train_epochs = 3	The total number of times the training dataset is passed through the model. Three epochs mean the entire dataset is used thrice for training the model, enhancing its learning.
train_batch_size = 8	The number of training examples processed together in one iteration. A batch size of 8 allows for a balanced trade-off between learning efficiency and memory usage.
eval_batch_size = 8	Similar to train_batch_size, but used during the model evaluation phase. It determines the data chunk size for each evaluation step.
Warmup_steps = 500	The initial steps where the learning rate gradually increases to its peak, reducing the risk of training instability in the early phases.
lora_model configuration	Adjustments specific to the LORA approach:
R = 32	The rank parameter for LORA, impacting the model’s approximation accuracy and the complexity of the adaptation.
Alpha = 64	Controls the scale of the Low-Rank Adaptation, influencing the balance between the original and adapted model components.
Lora_dropout = 0.05	The dropout rate used within the LORA adaptation to prevent overfitting by randomly omitting a fraction of the adaptation units during training.
Optimizer AdamW	Specifies the optimizer settings:
Adam-epsilon = 10^−8^	A small constant for numerical stability in the AdamW optimization algorithm.
Beta-1 = 0.9	The exponential decay rate for the first-moment estimates in AdamW, balancing the influence of past gradients.
Beta-2 = 0.98	The exponential decay rate for the second-moment estimates in AdamW, controlling the moving average of the squared gradients.

**Table 8:** Learning rate performance results.

Learning Rate	WER (%)	Normalized WER (%)	CER (%)	Normalized CER (%)	Training Loss	Validation Loss
$10^{-2}$	50.634	45.321	26.198	22.566	5.824	5.533
$10^{-3}$	25.781	18.774	15.337	12.103	0.378	0.510
$10^{-4}$	43.634	35.321	26.198	22.566	2.824	2.533

**Table 9:** Warmup steps performance results.

Warmup Steps	Normalized WER (%)	Normalized CER (%)	Training Loss	Validation Loss
250	21.563	18.235	0.499	0.667
500	18.774	15.337	0.288	0.510
750	18.720	15.349	0.270	0.499
1000	18.504	15.320	0.235	0.487
1250	23.757	19.813	0.507	0.689

**Table 10:** Model performance with different optimizer parameters.

Adam Epsilon	Normalized WER (%)	Training Loss	Validation Loss
$10^{-7}$	18.273	0.229	0.477
$10^{-9}$	17.640	0.219	0.457

**Table 11:** Model performance with different LORA configurations.

LORA Configuration (R)	Normalized WER (%)	Training Loss	Validation Loss
R = 42	17.660	0.223	0.467
R = 52	20.298	0.491	0.649

**Table 12:** Performance comparison across different Lora_dropout configurations.

Lora_Dropout	Normalized WER (%)	Training Loss	Validation Loss
0.05	17.866	0.263	0.491
0.06	19.176	0.413	0.556
0.04	17.121	0.210	0.448
0.03	19.968	0.472	0.589

## Data Availability

The data presented in this study are available on request from the corresponding author due to privacy.
